# Supplementary material for: Constrained brain volume in an efficient coding model explains the fraction of excitatory and inhibitory neurons in sensory cortices
Source: PLoS Comput Biol. 2022 Jan 21;18(1):e1009642. doi: 10.1371/journal.pcbi.1009642 (PMC8809590; doi:10.1371/journal.pcbi.1009642)
Supplement: S1 Text — Figure A: Estimation of Bias in Statistical Analysis. Figure B: Structure of Recurrent Inhibition. Figure C: Recurrent Excitation vs Inhibition (Normalized Activity Profiles). Figure D: Model Predictions (all measures) vs Biology. Figure E: Hierarchical Bootstrap. (PDF) [file pcbi.1009642.s001.pdf]

Supplementary Information: Constrained brain volume in an efficient coding model explains the fraction of excitatory and inhibitory neurons in sensory cortices

Arish Alreja[1], Ilya Nemenman[2], Christopher Rozell[3*]

[1] Neuroscience Institute, Center for the Neural Basis of Cognition and Machine Learning Department, Carnegie Mellon University, Pittsburgh, Pennsylvania, USA | [2] Department of Physics, Department of Biology and Initiative in Theory and Modeling of Living Systems, Emory University, Atlanta, Georgia, USA | [3] School of Electrical and Computer Engineering, Georgia Institute of Technology, Atlanta, Georgia, USA (*crozell@gatech.edu)

Supplementary Results

Bias in statistical analysis

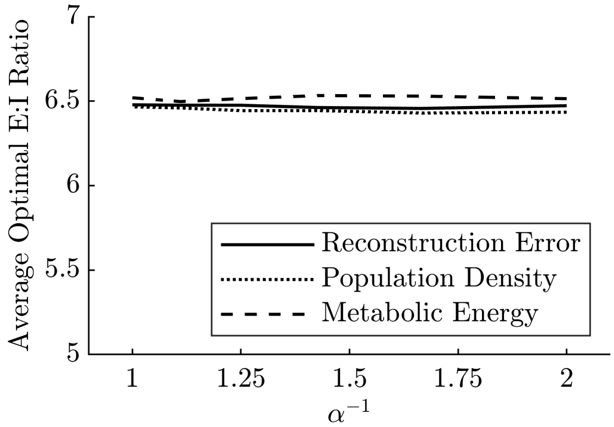

Figure A: **Estimation of Bias in Statistical Analysis:** Estimation of the bias involves choosing 100 patches from $N^* = \alpha N$, $\alpha < 1$ of the $N = 10$ natural images. Mean reconstruction error, population sparsity and metabolic energy consumption are computed for each E:I ratio. This constitutes a single run. For a given α, we perform 100 runs. This process is repeated for each $\alpha \in \{0.5, 0.6, 0.7, 0.8, 0.9, 1.0\}$, which amounts to a total of 600 runs. Average optimal E:I for $\lambda = 0.15$ and different values of α are shown for reconstruction error, population density and metabolic energy consumption. The relative constancy of average optimal E:I ratio over the bias runs for different values of α indicates that any possible bias in estimating the optimal E:I ratio is negligible for the explored sample sizes.

We estimated bias in all three performance measures using a bootstrap procedure (See *Supplementary Methods*) which samples from a subset of the natural image database [1] for models trained with a sparsity constraint of $\lambda = 0.15$. Fig A indicates stability of the mean optimal E:I ratio over 100 runs sampling from differently sized subsets (denoted by α) of the natural image databases, suggesting negligible bias.

Structure of Recurrent Inhibition

We examined the static structure of inhibition of the model at the optimal E:I ratio for different sparsity levels. We observed that inhibitory strength, represented by the Singular Values Σ, interpreted as implementing dendritic gain (see *Methods*) is distributed less evenly across the inhibitory sub-population as sparsity (λ) increases (Fig B.i), even as the total inhibitory strength/dendritic gain across the inhibitory sub-population remains relatively unchanged across different sparsity levels (Fig B.ii). Next, we use a metric called the stable rank [2] which is defined as

$$\text{Stable Rank} = \frac{\sum_{\mathrm{i}=1}^{\mathrm{N_i}} \sigma_{\mathrm{i}}^2}{\sigma_1^2}, \tag{1}$$

where σ_i is the ith singular value of the SVD of the recurrent matrix. The stable rank is relatively robust to smaller singular values. In the context of our interpretation of Σ as the dendritic gain, the stable rank can serve as an additional measure of unevenness (lower stable rank implies greater unevenness). The value of the stable rank decreases as sparsity (λ) increases (Fig

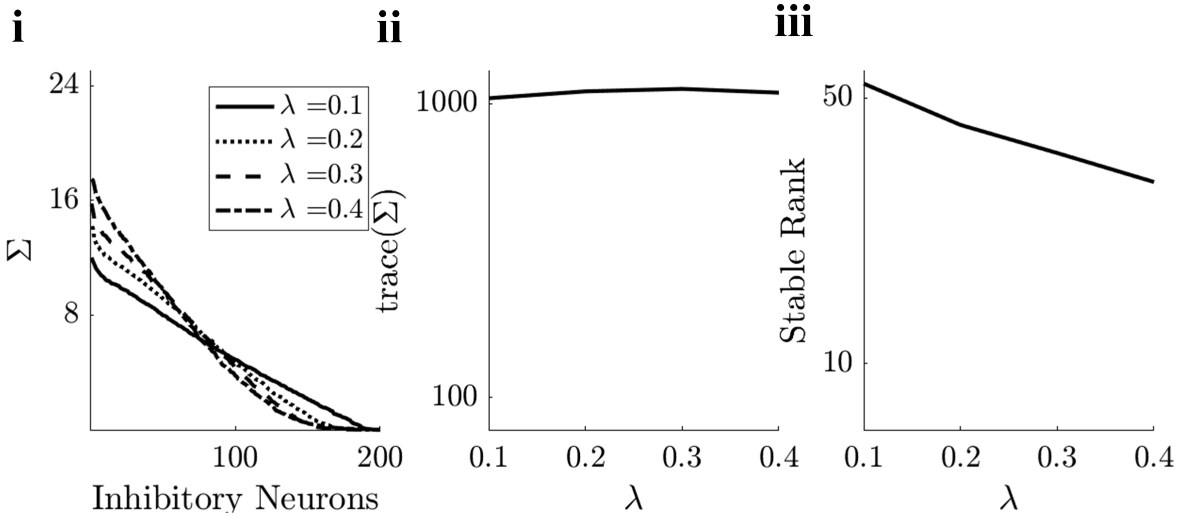

Figure B: **Structure of Recurrent Inhibition:** **(i)** Inhibitory strength Σ interpreted as being implemented via dendritic gain (see *Methods*) vs Inhibitory Interneurons (Components) for different sparsity levels (λ). **(ii)** Total amount of inhibitory strength/dendritic gain across the inhibitory sub-population, relatively unchanged for models at the optimal E:I ratio for different sparsity constraints (λ). **(iii)** The trend of increasing unevenness in inhibitory strength as sparsity (λ) increases, depicted by the first two plots is also reflected by a decrease in the stable rank measure detailed in [2].

B.iii) adding support for the preceding result that indicates that unevenness in inhibitory strength of the model at the optimal E:I ratio increases as sparsity increases. Together, these results suggest that while the total amount of inhibition supported by the structure is relatively unchanged for models at optimal E:I ratio at different sparsity levels, it is distributed less evenly in the inhibitory sub-population.

Inhibitory sub-population activity profiles at different sparsity levels

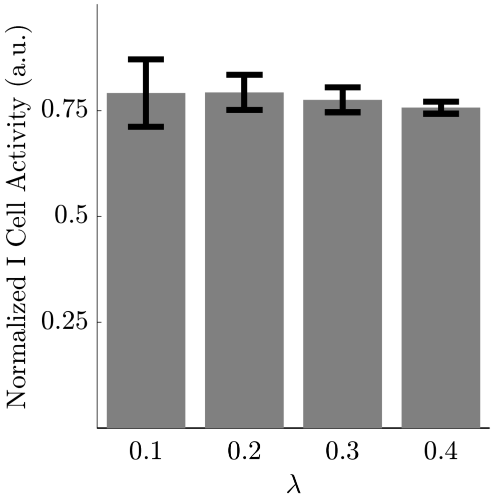

Figure C: **Recurrent Excitation vs Inhibition (Normalized Activity Profiles):** A normalized version of Fig. 3D shows I cell activity when normalized against the total (E+I cell) activity of the model. The normalized I cell activity is relatively unchanged across optimal models at different sparsity levels, while the diversity of responses (error bars) to different natural image stimuli in the inhibitory sub-population shows (like the un-normalized plot) that I cell responses are more specifically tuned to stimuli at lower sparsity levels/lower optimal E:I ratio and become broadly tuned and less diverse as model sparsity/optimal E:I ratio increases.

Model performance (all performance measures) vs biology

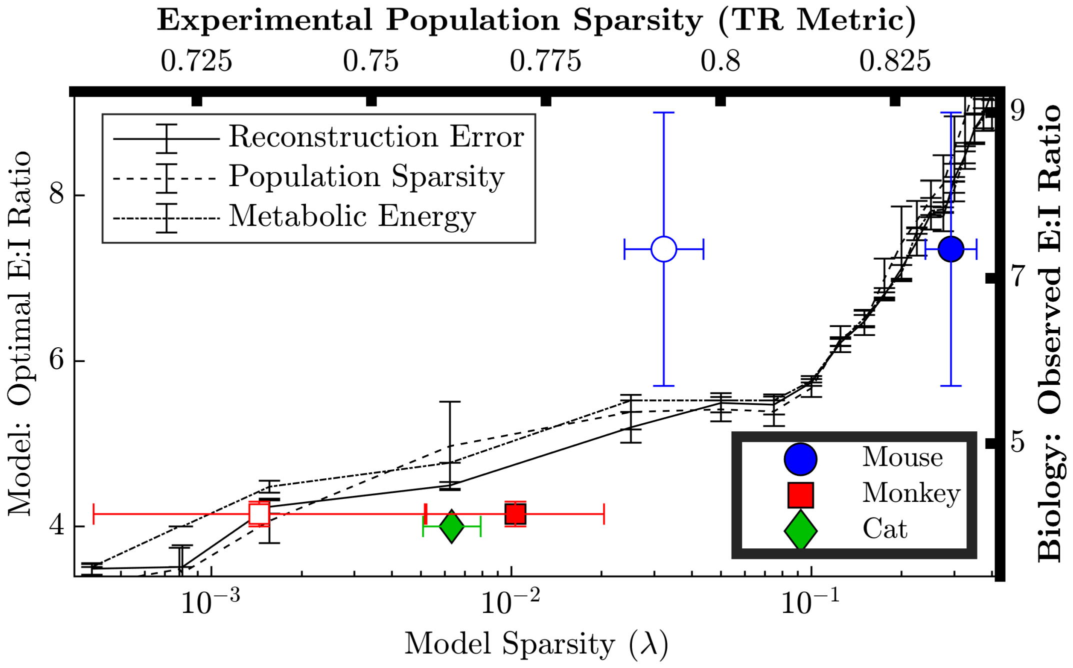

Figure D: **Model Predictions (all measures) vs Biology: (Left Y and Bottom X axes)** The Optimal E:I ratio as a function of model sparsity constraints λ in computational models according to all three performance measures is captured by the different lines with error bars denoting the standard error for each. A similar (but not identical) trend in the relationship between optimal E:I ratio and model sparsity is revealed for each of the 3 performance measures. **(Right Y and Top X axes)** The Population Sparsity (TR) measure computed for electrophysiology data from experimental studies in mice [3], monkeys [4,5] and cats [6] is shown as mean (markers) ± standard error(horizontal error bars) w.r.t. observed E:I ratio ranges (vertical error bars) in Biology with unfilled markers representing natural images and black filled markers representing natural movies.

Supplementary Methods

Estimation of statistical errors in the analysis

We evaluate variance and bias in reconstruction error, population sparsity and metabolic energy over the image patch database of 10 natural images from which image patches are sampled at each E:I ratio (1:1-10:1).

To estimate the variance, we randomly select 1 out of $N = 10$ images (with replacement) and we select 10 16x16 pixel image patches from this image.Variance is estimated for the sparsity constraints $\lambda \in \{0.1,0.15,0.2,0.25,0.3\}$. Repeating this process 10 times, we gather 100 16x16 pixel image patches. We perform inference in the model and calculate the mean reconstruction error, population sparsity and metabolic energy consumption are computed using the model corresponding to each E:I ratio. This constitutes one run. We collect and aggregate statistics from 100 runs. The standard deviation of the means computed for each of the runs is the standard error for a given performance measure for a given model.

Estimation of the bias is similar, however, instead of choosing patches from $N = 10$ images, we use N^* images, where $N^* = \alpha N$, $\alpha < 1$. Bias is estimated for sparsity constraint λ=0.15. Mean reconstruction error, population sparsity and metabolic energy consumption are computed for each E:I ratio. This constitutes a single run. For a given α, we perform 100 runs. We repeat this process for each $\alpha \in \{0.5, 0.6, 0.7, 0.8, 0.9, 1.0\}$ ($\alpha = 1.0$ being the variance estimate mentioned above), which amounts to a total of 600 runs. The average (over 100 runs) optimal E:I ratio for $\lambda = 0.15$ at different values of α is then examined to explore if the image patch database size induces any bias in the observed optimal E:I ratio.

Estimation of the ratio of recurrent excitation vs recurrent inhibition during stimulus representation

To better understand the effects of changes in the size of inhibitory subpopulation (i.e., different E:I ratios), we examine the relationship between recurrent excitation and recurrent inhibition received by active units in response to stimulus image patches. We consider the following decomposition of the low rank approximation (G) of the recurrent connectivity matrix utilized in an earlier study [7]

$$\mathrm{G} = \underbrace{U^{+}\Sigma V^{-} + U^{-}\Sigma V^{+}}_{G_{excite}} + \underbrace{U^{+}\Sigma V^{+} + U^{-}\Sigma V^{-}}_{G_{inhib}}. \tag{2}$$

The recurrent excitation and inhibition received by active nodes is computed as

$$\text{Recurrent E} = [(D - G_{excite}) \times a] \circ \mathbb{I}_{a>0}, \tag{3}$$

$$\text{Recurrent I} = [G_{inhib} \times a] \circ \mathbb{I}_{a>0}, \tag{4}$$

where $\mathbb{I}$ is the standard indicator function taking the value 1 if the argument is true and 0 otherwise. We compute a ratio between recurrent excitation and recurrent inhibition received by active neurons as

$$\text{Recurrent Ratio} = \frac{\text{Recurrent E}}{\text{Recurrent I}}. \tag{5}$$

The Recurrent Ratio is computed for each anatomical E:I ratio for models with different sparsity constraints (λ). Here we evaluate the recurrent E/I balance specifically in response to stimulus, and recognize that the network is more stable when recurrent inhibition is greater than recurrent excitation.

Hierarchical bootstrap: Hypothesis testing model predictions against multi-level experimental data

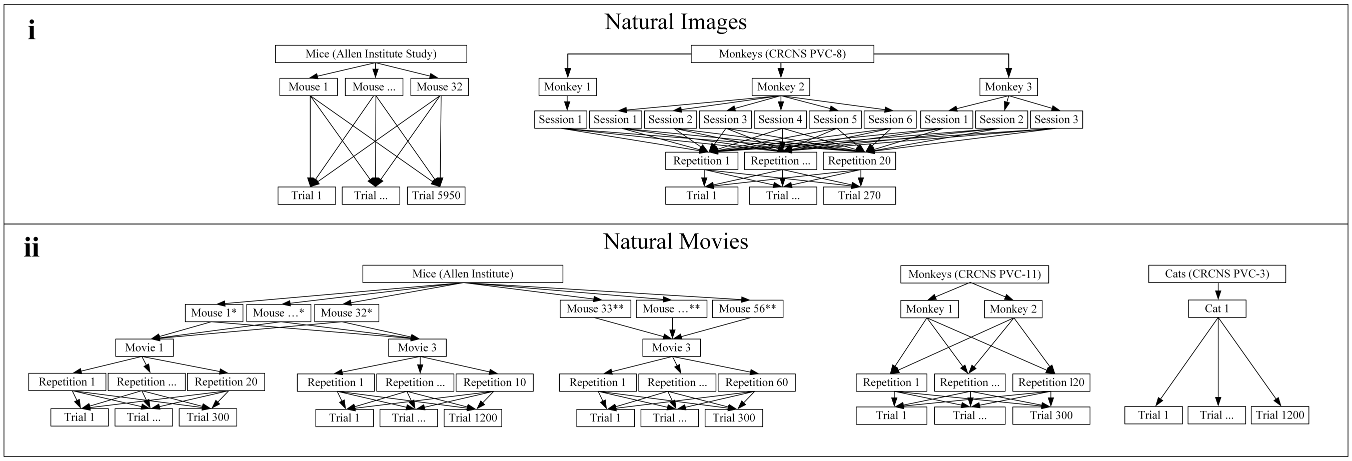

Figure E: **Hierarchical Bootstrap:** The hierarchy of multi-level experimental data sets used to draw comparisons between population sparsity of different species is represented in each figure for different stimulus types. **(i)** For natural images, the available data sets allow us to draw a comparison between 32 mice (E:I = 5.7-9:1) [3] with 44-244 V1 neurons and 3 monkeys (E:I = 4-4.3:1) [5] with 16-76 V1 neurons. The monkey data [5] features multiple recording sessions per subject (1,6,3 sessions for monkeys 1,2,3) and 20 repetitions of all stimuli images in a block structure. **(ii)** For natural movies, the available data sets allow us to draw a comparison between 56 mice [3] with 44-244 V1 neurons, 2 monkeys [4] with 69-104 V1 neurons and 1 cat [6] (E:I = 4:1) with 10 V1 neurons. The mouse subjects come from 2 different experiments (* denotes the Brain Observatory Experiment with 32 subjects, and ** denotes the Functional Connectivity Experiment with 24 subjects) where the key differences include the number of different natural movies shown and how often each movie is repeated.

The hierarchical bootstrap procedure is built around sampling with replacement at different levels of a hierarchy in a multi-level dataset at each bootstrap run to estimate averages. The procedure is described in detail in [8].

Our measure of interest in this study is the scalar population sparsity measure (TR metric), which means that the number of recorded neurons don't feature in our hierarchy. As an example specific to its usage in this study, we describe the case of the

hierarchical bootstrap for comparing average population sparsity between mice and monkeys for natural image stimuli from multi-level data sets visualized in Fig E, to test the hypothesis/model prediction that mice should exhibit higher population sparsity than monkeys. For computational efficiency, population sparsity is pre-computed for each dataset before the hierarchical bootstrap procedure.

For mice, we first sample subjects (first level of hierarchy) with replacement from the 32 mice with stimulus responses to natural images in the Allen Institute data set [3]. Next, for each sampled mouse, we sample trials (second level of hierarchy) with replacement from the total number of trials 'T' (T=5950 in the example). Finally, we average the population sparsity across all the sampled trials to obtain an average population sparsity for natural images in mice. This process represents a single bootstrap run. A slightly different hierarchy, shown in Fig E.i is constructed for monkeys, where the first level represents different recording sessions (with different numbers of neurons) for a single monkey. The same hierarchical bootstrap procedure is repeated. We collect average population sparsity estimates for mice and monkeys for a total of 10,000 bootstrap runs for natural images as well as other stimulus types.

For hypothesis testing related to the example above, we treat the 10,000 average population sparsity values for mice and monkeys as a 2 dimensional distribution. We use this joint distribution to evaluate the model hypothesis/prediction that population sparsity in mice (E:I = 5.7-9:1) should be higher than monkeys (E:I = 4-4.3:1). With mice on the x-axis and monkeys on the y-axis, we compute the volume of the distribution where $x > y$ (i.e. the volume of the distribution below the line $y = x$). If the volume $> 1 - \frac{\alpha}{2}$ then mouse pop sparsity > monkey pop sparsity at level α, ($\alpha = 0.05$ in our analysis). In the event of multiple comparisons (e.g. natural movie stimulus), Bonferroni (or other) corrections can be applied the same way as traditional hypothesis testing. The volume of the distribution opposing the hypothesis (above $y = x$ in our example) is the p value for the test. It is referred to as $p_{bootstrap}$ to disambiguate it from p values emanating from traditional hypothesis testing.

References

1. Olshausen BA, Field DJ. Emergence of simple-cell receptive field properties by learning a sparse code for natural images. Nature. 1996;381(6583):607–609.

2. Eftekhari A, Yap HL, Wakin MB, Rozell CJ. Stabilizing embedology: Geometry-preserving delay-coordinate maps. Physical Review E. 2018;97(2):022222.

3. Siegle JH, Jia X, Durand S, Gale S, Bennett C, Graddis N, et al.. Data from ”A survey of spiking activity reveals a functional hierarchy of mouse corticothalamic visual areas”; 2019. bioRxiv. Available from: `https://www.biorxiv.org/content/early/2019/10/16/805010`.

4. Kohn A, Smith MA. Data from ”Utah array extracellular recordings of spontaneous and visually evoked activity from anesthetized macaque primary visual cortex (V1).”; 2016. CRCNS.org.

5. Kohn A, Coen-Cagli R. Data from ”Multi-electrode recordings of anesthetized macaque V1 responses to static natural images and gratings.”; 2015. CRCNS.org.

6. Blanche T. Data from ”Multi-neuron recordings in primary visual cortex.”; 2009. CRCNS.org.

7. Zhu M, Rozell CJ. Modeling biologically realistic inhibitory interneurons in sensory coding models. PLoS Computational Biology. 2015;11(7):e1004353.

8. Saravanan V, Berman GJ, Sober SJ. Application of the hierarchical bootstrap to multi-level data in neuroscience; 2019.

Legends

1. Supplementary Results

   (a) Figure A: **Estimation of Bias in Statistical Analysis:** Estimation of the bias involves choosing 100 patches from $N^* = \alpha N$, $\alpha < 1$ of the $N = 10$ natural images. Mean reconstruction error, population sparsity and metabolic energy consumption are computed for each E:I ratio. This constitutes a single run. For a given α, we perform 100 runs. This process is repeated for each $\alpha \in \{0.5, 0.6, 0.7, 0.8, 0.9, 1.0\}$, which amounts to a total of 600 runs. Average optimal E:I for $\lambda = 0.15$ and different values of α are shown for reconstruction error, population density and metabolic energy consumption. The relative constancy of average optimal E:I ratio over the bias runs for different values of α indicates that any possible bias in estimating the optimal E:I ratio is negligible for the explored sample sizes.

   (b) Figure B: **Structure of Recurrent Inhibition: (i)** Inhibitory strength Σ interpreted as being implemented via dendritic gain (see *Methods*) vs Inhibitory Interneurons (Components) for different sparsity levels (λ). **(ii)** Total amount of inhibitory strength/dendritic gain across the inhibitory sub-population, relatively unchanged for models at the optimal E:I ratio for different sparsity constraints (λ). **(iii)** The trend of increasing unevenness in inhibitory strength as sparsity (λ) increases, depicted by the first two plots is also reflected by a decrease in the stable rank measure detailed in [2].

   (c) Figure C: **Recurrent Excitation vs Inhibition (Normalized Activity Profiles):** A normalized version of Fig. 3D shows I cell activity when normalized against the total (E+I cell) activity of the model. The normalized I cell activity is relatively unchanged across optimal models at different sparsity levels, while the diversity of responses (error bars) to different natural image stimuli in the inhibitory sub-population shows (like the un-normalized plot) that I cell responses are more specifically tuned to stimuli at lower sparsity levels/lower optimal E:I ratio and become broadly tuned and less diverse as model sparsity/optimal E:I ratio increases.

   (d) Figure D: **Model Predictions (all measures) vs Biology: (Left Y and Bottom X axes)** The Optimal E:I ratio as a function of model sparsity constraints λ in computational models according to all three performance measures is captured by the different lines with error bars denoting the standard error for each. A similar (but not identical) trend in the relationship between optimal E:I ratio and model sparsity is revealed for each of the 3 performance measures. **(Right Y and Top X axes)** The Population Sparsity (TR) measure computed for electrophysiology data from experimental studies in mice [3], monkeys [4,5] and cats [6] is shown as mean (markers) $\pm$ standard error(horizontal error bars) w.r.t. observed E:I ratio ranges (vertical error bars) in Biology with unfilled markers representing natural images and black filled markers representing natural movies.

2. Supplementary Methods

   (a) Figure E: **Hierarchical Bootstrap:** The hierarchy of multi-level experimental data sets used to draw comparisons between population sparsity of different species is represented in each figure for different stimulus types. **(i)** For natural images, the available data sets allow us to draw a comparison between 32 mice (E:I = 5.7-9:1) [3] with 44-244 V1 neurons and 3 monkeys (E:I = 4-4.3:1) [5] with 16-76 V1 neurons. The monkey data [5] features multiple recording sessions per subject (1,6,3 sessions for monkeys 1,2,3) and 20 repetitions of all stimuli images in a block structure. **(ii)** For natural movies, the available data sets allow us to draw a comparison between 56 mice [3] with 44-244 V1 neurons, 2 monkeys [4] with 69-104 V1 neurons and 1 cat [6] (E:I = 4:1) with 10 V1 neurons. The mouse subjects come from 2 different experiments (* denotes the Brain Observatory Experiment with 32 subjects, and ** denotes the Functional Connectivity Experiment with 24 subjects) where the key differences include the number of different natural movies shown and how often each movie is repeated.
